# Exploring the relationship between environmental drivers and the manifestation of fibropapillomatosis in green turtles (*Chelonia mydas*) in eastern Brazil

**DOI:** 10.1371/journal.pone.0290312

**Published:** 2023-08-24

**Authors:** Ralph E. T. Vanstreels, Alexis Durant, Allan P. Santos, Robson G. Santos, Angélica M. S. Sarmiento, Silmara Rossi, Fabiola E. Setim, Marco A. Gattamorta, Eliana R. Matushima, Luis F. S. P. Mayorga, Marcela M. Uhart

**Affiliations:** 1 Karen C. Drayer Wildlife Health Center, School of Veterinary Medicine, University of California, Davis, CA, United States of America; 2 Instituto de Pesquisa e Reabilitação de Animais Marinhos, Cariacica, ES, Brazil; 3 Faculdade de Medicina Veterinária e Zootecnia, Departamento de Patologia, Laboratório de Patologia Comparada de Animais Selvagens, Universidade de São Paulo, São Paulo, SP, Brazil; 4 Laboratório de Biologia Marinha e Conservação, Universidade Federal de Alagoas, Maceió, AL, Brazil; 5 Instituto Argonauta para a Conservação Costeira e Marinha, Ubatuba, SP, Brazil; 6 Projeto Cetáceos da Costa Branca, Universidade do Estado do Rio Grande do Norte, Areia Branca, RN, Brazil; 7 Universidade São Judas, São Paulo, SP, Brazil; 8 Universidade Paulista, São Paulo, SP, Brazil; 9 Instituto Federal de São Paulo, São Paulo, SP, Brazil; MARE – Marine and Environmental Sciences Centre, PORTUGAL

## Abstract

Fibropapillomatosis (FP) is a disease characterized by epithelial tumors that can impede life-sustaining activities of sea turtles, especially green turtles (*Chelonia mydas*). FP is caused by a herpesvirus, but environmental factors are also thought to play a role in triggering FP tumor growth. In this study, we evaluate the epidemiology of FP tumors in green turtles along the coast of Espírito Santo, Brazil, a region where juvenile green turtles are known to aggregate with high FP prevalence. A dataset comprising 2024 beach-cast green turtles recorded through daily beach surveys on 400 km of coastline from 2018 to 2021 (inclusive) was evaluated. FP tumors were recorded in 40.9% of the individuals in this dataset, and presence of FP tumors was predicted by individual variables (presence of marine leeches, stranding code, curved carapace length, body mass-size residual) and characteristics of the stranding site (distance to nearest metallurgical plant, mean sea surface salinity (SSS), annual range of sea surface temperature (SST)). Additionally, a second dataset comprising detailed information about the size and anatomical distribution of tumors in 271 green turtles with FP from the same region was evaluated. Hierarchical clustering analysis revealed these turtles could be classified in three groups according to the anatomical distribution of their tumors, and in turn the group to which each turtle was assigned could be predicted by the study period (2010–2014 vs. 2018–2022) and by characteristics of the stranding/capture site (green turtle stranding density, mean sea surface chlorophyll-a concentration, mean SSS, mean SST, annual range of SST). These results corroborate that individual and environmental factors play a significant role driving FP epidemiology. Furthermore, the results suggest that rather than behaving as a single entity, FP may be seen as a mosaic of distinct anatomical patterns that are not necessarily driven by the same environmental factors.

## Introduction

Green turtles (*Chelonia mydas*) are distributed throughout tropical and subtropical seas worldwide and are classified as “endangered” [1]. They are important members of their ecosystems, playing a role in the transfer of nutrients from marine to terrestrial ecosystems, participating in coastal and pelagic trophic webs as consumers, prey and predators, and serving as substrate for the long-distance transfer of epibionts [2,3]. The coastal waters of eastern Brazil are important juvenile foraging grounds for green turtles hatched in the South Atlantic Ocean [4,5] however fisheries bycatch, habitat degradation, and fibropapillomatosis (FP) are important threats in this region [1,5].

Fibropapillomatosis is a neoplastic disease impacting sea turtles globally, causing epithelial tumors that can impede life-sustaining activities [6,7]. FP tumors are generally external, and can vary considerably in number, size, pigmentation, texture, and location [6–8]. The disease was first described in 1938 and remained at a low prevalence until the number of cases expanded rapidly worldwide in the 1980s [9]. Although FP has been reported in all seven species of sea turtles, juvenile green turtles are by far the most affected both in terms of prevalence and severity [7]. *Chelonid herpesvirus 5* (ChHV5) is widely accepted as the causative agent of FP, since the transmissibility of tumors was experimentally demonstrated with evidence of a herpesvirus agent [6,10–12] and by the *de novo* formation of ChHV5-positive intranuclear inclusions in cultured green turtle cells [13]. However, epidemiological studies suggest that ChHV5 infection is necessary, but not sufficient, for FP tumor development [7]. ChHV5 is frequently present in the skin and other tissues of sea turtles without tumors, suggesting that additional factors are necessary to trigger tumor development in infected individuals [14–17]. Because several studies have demonstrated that FP prevalence is usually higher in areas of reduced water quality, a variety of environmental factors have been proposed to play a role directly or indirectly in driving tumor manifestation [6,7,18–22]. Coinfection by papillomavirus has also been recently proposed as a secondary trigger for FP tumor development [23].

Strictly speaking, FP tumors are histologically benign, but the multifocal distribution of the tumors in many cases suggest the potential for hematogenous, lymphatic, and/or neuronal spread throughout the body. However, depending on their size and anatomic location they can produce negative health outcomes by interfering with vision, locomotion, ability to eat, reproduction, and predator avoidance [7,11,24,25]. External tumors tend to be most abundant on the anterior parts of the body, especially on the front flippers, a pattern consistently observed in green turtles worldwide [8,22,24,26–28]. On the other hand, while ocular tumors are generally frequent in Indonesia [26], Hawaii [24], and Lesser Antilles [27], they are relatively uncommon in Brazil [8,28]. Furthermore, although oral and visceral tumors are relatively common in Hawaii and Florida [15,24,29,30], they are scarcely reported elsewhere [8,22,28,31].

**Fig 1:**
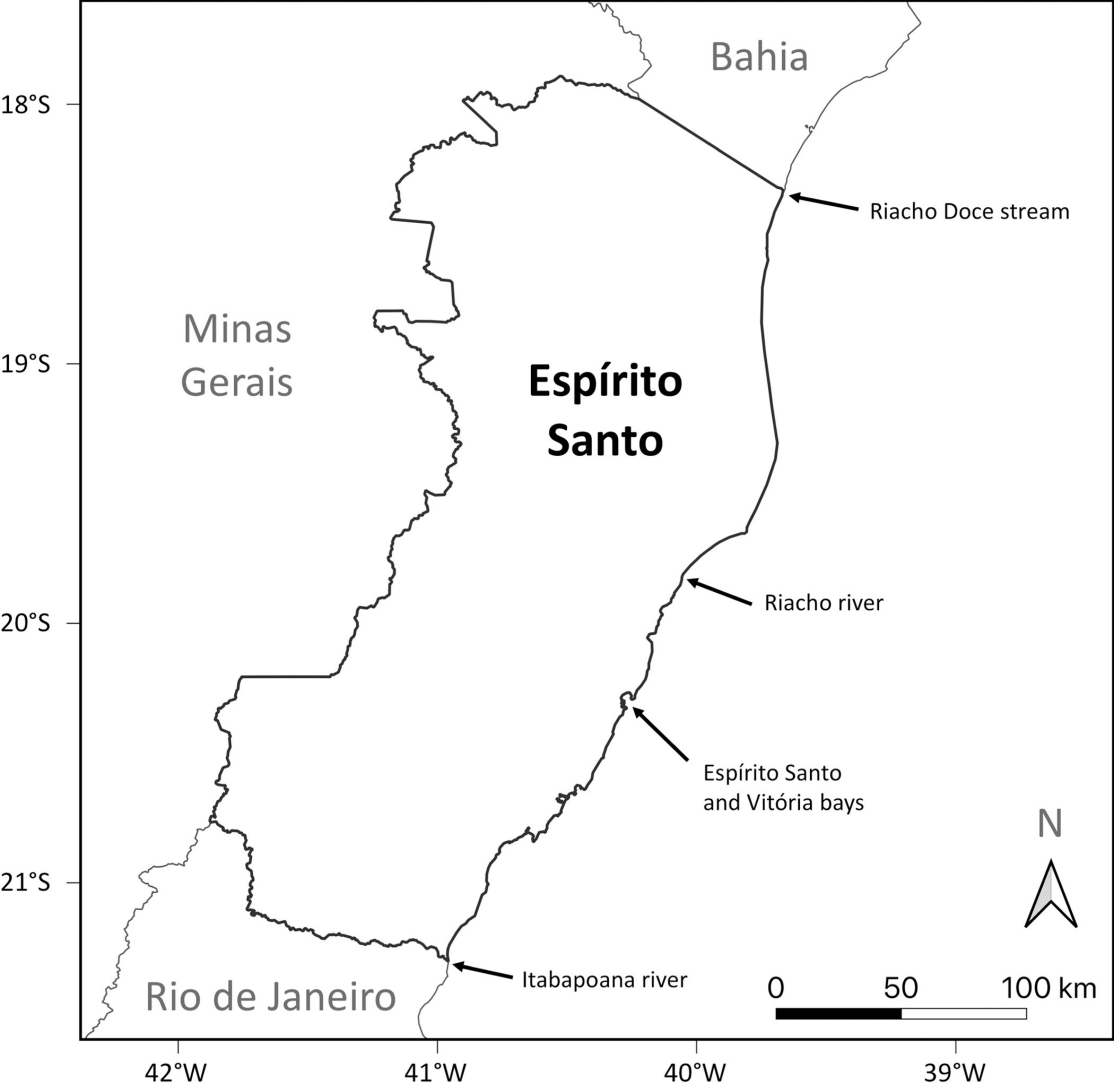
Location of the study area (Espírito Santo, Brazil) and landmarks mentioned in the text.

The state of Espírito Santo, located in the southeast region of Brazil (Fig 1), is considered a hotspot for FP in the Southwest Atlantic Ocean [28,32]. Surveys at areas of aggregation of juvenile green turtles in Espírito Santo have reported FP prevalence of 58%, with an average of 40 tumors per affected individual [18]. While the reason for this high prevalence is unknown, it has been suggested that reduced water quality from urban centers and thermal pollution by coastal metallurgical industries might play a role [18,33–35]. Despite the high prevalence, FP has been far less studied in Brazil compared to sites in the northern hemisphere [36]. In this study, we analyze two large datasets on the occurrence, size, and anatomical distribution of tumors on green turtles in Espírito Santo. Dataset 1 comprises information on green turtles found ashore on the coast of Espírito Santo through consistent daily beach surveys from 2018 to 2021; this dataset has information on the presence or absence of external FP tumors, but does not provide details about their size or anatomical distribution. Dataset 2 comprises information on a subset of green turtles with FP from Dataset 1 (beach-cast turtles from 2018 to 2021) as well as previously-published data on green turtles with FP from the same area (a combination of beach-cast, by-caught and intentionally captured turtles from 2010 to 2014); this dataset has detailed information on the size or anatomical distribution of external FP tumors. The combined interpretation of these datasets provides an opportunity to unravel the epidemiology of FP and to evaluate the potential relationships between individual and environmental variables and the occurrence and anatomical distribution of FP tumors.

## Methods

This study was approved by the Animal Use Ethics Committee of the School of Veterinary Medicine and Animal Science of University of São Paulo (CEUA 697/2005, 1932/2010, 2116/2010, 2555/2012). Permits were obtained from the Authorization and Information System in Biodiversity of the Chico Mendes Institute for Biodiversity Conservation of the Brazilian Ministry of the Environment (SISBIO 21802, 22751, 26667, 26896, 32636).

### Study area

The study area corresponds to the coastline of Espírito Santo state (Fig 1), extending approximately 400 km from Riacho Doce stream (18.35S, 39.67 W) to Itabapoana river (21.31S, 40.96 W). This region represents the southwestern limit of the core distribution of green turtles in the Atlantic Ocean, and is characterized by oligotrophic tropical-subtropical transitional waters of the eastern Brazil ecoregion in the Tropical Southwestern Atlantic marine province [37,38]. Espírito Santo coastal waters are an important feeding ground for juvenile green turtles, with most individuals presenting curved carapace length (CCL) between 30 and 65 cm [39]. Juvenile green turtles foraging along the coast of Espírito Santo are predominantly of mtDNA haplotypes CM-A8 (56%) and CM-A5 (30%) [4], the most common and widespread haplotypes in Atlantic equatorial rookeries [40,41]. Mark-recapture and satellite tracking studies have shown that Espírito Santo is an important foraging area for juveniles born in Ascension Island [42,43]. The overall prevalence of FP in green turtles in Espírito Santo was estimated at 27.4% [28]. Warm water effluent discharge from metallurgical (iron ore/steel) plants is known to attract aggregations of juvenile green turtles in this area, and underwater surveys have reported average FP prevalence between 34.4% and 75.8% at these sites [18,33–35]. At least two closely-related ChHV5 variants occur in juvenile green turtles with FP in Espírito Santo, both of which are similar to those that circulate in the South Atlantic Ocean but are distinct from the variants found in the Caribbean and Pacific Ocean [44].

### Dataset 1: Fibropapillomatosis tumor prevalence

Since 2010, the Espírito Santo coast is part of the Beach Monitoring Project of the Campos and Espírito Santo basins (Projeto de Monitoramento de Praias da Bacia de Campos-Espírito Santo, PMP-BC/ES). PMP-BC/ES is one of the monitoring programs required by Brazil’s federal environmental agency, IBAMA, for the environmental licensing of oil production and transport by Petrobras. Through PMP-BC/ES, live and dead marine tetrapods found ashore during daily beach surveys (by foot or quadricycle) or reported by the public (through a toll-free telephone line; may include beach-cast animals as well as fisheries bycatch) on the entire coast of Espírito Santo (Fig 1) are photographed and recorded following standardized protocols [45]; live individuals are provided veterinary care and dead individuals in reasonable preservation conditions are necropsied. Since September 2017, the data produced through PMP-BC/ES is publicly available through the SIMBA platform (Sistema de Informação de Monitoramento da Biota Aquática, http://simba.petrobras.com.br/).

We evaluated data from 6755 green turtles recorded by the PMP-BC/ES along the Espírito Santo coast from January 2018 to December 2021. The following information about environmental conditions at the time of collection in the field was recorded for each turtle: date, geographic coordinates, weather conditions (clear, partly cloudy, cloudy, rainy, foggy), Douglas sea scale (score from 0 to 9 according to wave height), tide (rising, high, falling, low), Beaufort wind force scale (score from 0 to 12 according to wind speed), and wind direction (north, northeast, east, southeast, south, southwest, west, northwest). When feasible, the body mass and CCL of each individual was recorded. Following PMP-BC/ES protocol [45], each animal was assigned a code according to its level of preservation (S1 Table): 1 (live animal), 2 (fresher carcass), 3 (carcass in moderate decomposition, but organs basically intact), 4 (carcass in advanced decomposition), or 5 (mummified or skeletal remains). Live individuals (code 1) were transferred to a rehabilitation facility, where a clinical report was produced; if these animals died while under care, their carcasses were subjected to necropsy. Necropsied carcasses were further evaluated according to a standardized protocol where the sex was determined (male, female, not determined) and the presence or absence of evidence of fishery bycatch was recorded.

Descriptive text fields from field records, clinical reports and necropsy reports were searched for terms that could indicate the presence of tumors (search terms in Portuguese: tumor*, fibrom*, fibropapilom*, papilom*, massa, neoplasia, deformidade, verrucos*), marine leeches (sanguessuga, ectoparasit*, placa, ovo, larva), and epibionts (epibiont*, alga, craca, encrustad*, poliquet*, verde, vermelh*, marrom). To determine if FP tumors, marine leeches (*Ozobranchus* sp.), or epibionts (barnacles, algae, polychaetes, etc.) were present or absent, any text containing these search terms was thoroughly read and analyzed. Through this procedure, it was determined that the written records of 6755 turtles (100%) had explicit information about the presence/absence of tumors, 5464 turtles (80.9%) had explicit information about marine leeches, and 6024 turtles (89.2%) had explicit information about epibionts. Because data available through SIMBA were produced by a multitude of individuals with varying levels of expertise and diligence, we conducted a systematic assessment of data quality. For this purpose, photographs from 676 individuals (10% of the dataset) were downloaded and visually inspected in order to determine whether the presence or absence of tumors and marine leeches had been correctly reported in text. This analysis revealed that the presence of tumors had been incorrectly reported for 0.3% of turtles (2 false negatives), the presence of marine leeches had been incorrectly reported for 3.1% of turtles (19 false positives, 2 false negatives), and the presence of epibionts had been incorrectly reported for 10.1% of turtles (8 false positives, 71 false negatives). Based on these results, it was determined that data regarding tumors and marine leeches had acceptable quality, whereas data about epibionts were not sufficiently reliable and had to be excluded from further analyses.

On a second stage of data quality checking, the prevalence of FP tumors was compared among stranding codes to determine whether FP prevalence might be underestimated in carcasses that were recovered in an advanced stage of decomposition (S2 Table). The prevalence of FP tumors was markedly lower in carcasses of code 4 (14.4%; 548/3796) and code 5 (4.0%; 37/935) compared to live turtles (41.3%; 187/453) and carcasses of code 2 (49.4%; 78/158) and code 3 (39.8%; 563/1413). Our experience suggests this difference was likely related to the difficulty of recognizing FP tumors in heavily degraded carcasses. Hence, carcasses of codes 4 and 5 were excluded from further analyses, resulting in a final sample size of 2024 turtles.

### Dataset 2: Anatomical distribution of fibropapillomatosis tumors

Detailed data on tumor size and anatomical distribution were evaluated from 271 green turtles with FP. For logistical reasons, this dataset focused on the southern portion of the study area, extending approximately 250 km from Riacho river (19.83S, 40.06 W) to Itabapoana river (Fig 1). This dataset comprised 142 beach-cast green turtles collected through PMP-BC/ES from 2018 to 2022 (individuals also in dataset 1), of which 51 were live individuals and 91 carcasses. Additionally, data were obtained on 129 green turtles studied by Rossi and colleagues [8] from 2010 to 2014, comprising 29 live-caught turtles (20 captured by researchers using casting nets, 9 bycaught in fishing nets), 5 live beach-cast individuals, and 95 beach-cast carcasses. All individuals in this dataset were juveniles, with CCL ranging from 25.6 to 74.0 cm (mean ± SD = 41.4 ± 6.1 kg) and body mass ranging from 1.48 to 42 kg (7.63 ± 4.40 kg).

The following information was collected for each turtle in this dataset: date, geographic coordinates, status (live capture, live rescue, beach carcass), CCL, and body mass. External tumors were classified into size categories according to their greater width [46]: A (<1 cm), B (1–4 cm), C (>4–10 cm), and D (>10 cm). Tumors of each size category were counted separately for each of eight anatomical regions: eyes, head, neck, forelimbs, carapace, plastron, hindlimbs, and inguinal/tail region [8]. The classification and counting of tumors were done in person by the authors (R.G.S., A.P.S., R.E.T.V., S.R., A.M.S.S., M.A.G., and F.E.S.) to ensure data consistency and reliability. The fibropapillomatosis index (FPI) was calculated for each turtle and for each anatomical region of each turtle using the formula: FPI = 0.1 × N_A_ + 1 × N_B_ + 20 × N_C_ + 40 × N_D_, where N_X_ corresponds to the number of tumors in the X size category [8]. Then, the relative FPI contribution of each anatomical region was calculated as the proportion of the individual’s total FPI corresponding to that anatomical region. Each turtle was classified according to the Southwest Atlantic fibropapillomatosis score (FPS_SWA_): mild (FPI < 40), moderate (40 ≤ FPI < 120), or severe (FPI ≥ 120) [8]. Unfortunately, no data were available on the occurrence of marine leeches in the individuals sampled during 2010–2014, hence this variable could not be included in the analyses.

### Body mass-size residuals

Polynomial linear regression analysis was used to determine the relationship between CCL and body mass using data from 2395 green turtles (individuals from dataset 1), with CCL ranging from 25.4 to 127.0 cm and body masses ranging from 0.95 to 213.3 kg. Body mass was predicted by the following formula (S = 2.608, R^2^ = 0.949): Mass = 24.05 + 1.419 × CCL—0.002532 × CCL^2^ + 0.000225 × CCL^3^. A percentage body mass-size residual index (BSMR) was then calculated for each turtle in this study by subtracting the predicted body mass from the measured body mass, and dividing the result by the predicted body mass [47].

### Environmental variables

Environmental variables considered in this study are represented in S1 File. ArcGIS Pro (Environmental Systems Research Institute–Redlands, USA) was used to process geographic information. Green turtle stranding density was calculated as the number of turtle strandings recorded in daily beach surveys from 2018 to 2021 within each 10-km buffer polygon. Polygons of protected areas were obtained from the Brazilian Ministry of Environment (https://antigo.mma.gov.br/areas-protegidas/cadastro-nacional-de-ucs/dados-georreferenciados.html) and used to determine whether the stranding site of each turtle was within a protected area (tolerance = 100 meters). Polygons of bays were manually drawn for any areas where the coastline had a concave shape exceeding 3 km in width and used to determine whether the stranding site of each turtle was within a bay (tolerance = 100 meters). Polylines of major rivers were obtained from the DIVA-GIS dataset (https://www.diva-gis.org/) and used to produce points for each river mouth in Espírito Santo, which were then used to calculate the distance of the stranding site of each turtle relative to the nearest river mouth. The point location of the metallurgical plants in Espírito Santo was derived from the list of associates of the Union of Metallurgical and Electrical Material Industries of the State of Espírito Santo (https://www.sindiferes.com.br/) and based on information from previous site visits by the authors; these points were then used to calculate the distance of the stranding site of each turtle relative to the nearest metallurgical plant.

A 10-km buffer polygon was obtained for each turtle by drawing a 10-km radius circle polygon centered at the capture/stranding site. The average values within these 10-km buffer polygons were obtained for human population density (Gridded Population of the World, https://sedac.ciesin.columbia.edu/data/collection/gpw-v4), mean surface chlorophyll-a concentration (CHL) (Mean Annual Sea Surface Chlorophyll-a Concentration 2009–2013, https://data.unep-wcmc.org/datasets/37), and mean and annual range of the sea surface salinity (SSS) and temperature (SST) (MARSPEC, http://www.marspec.org/).

### Statistical analyses

Statistical analyses were conducted with R 4.2.1 [48] with the packages cluster 2.1.4 [49], effects 4.2–2 [50], factoextra 1.0.7 [51], MASS 7.3–57 [52], nnet 7.3–17 [53], and pscl 1.5.5 [54].

For dataset 1, binomial logistic regression (BLR) models were used to determine whether the presence of FP tumors (logical variable: true or false) on a turtle could be predicted by the following variables: year (as a number, including day and month as decimals), stranding environment (factor; categories “mangrove” and “other” were combined due to low sample size), weather conditions (factor; categories “rainy” and “foggy” were combined due to low sample size), Douglas sea scale (integer), tide (factor), Beaufort wind force scale (integer), wind direction (factor), stranding code (factor), CCL (cm), BMSR (percentage), marine leeches (logical), fishery bycatch (logical), protected area (logical), bay (logical), distance to nearest river mouth (km), distance to nearest metallurgical plant (km), turtle stranding density within 10 km (integer), mean human population density within 10 km (inhabitants/km^2^), mean CHL within 10 km (mg/m^3^), mean SSS within 10 km (‰), mean annual range of SSS within 10 km (‰), mean SST within 10 km (°C), and mean annual range of SST within 10 km (°C). To account for ontogenetic changes, CCL and BMSR were included as quadratic effects (ax^2^+bx).

For dataset 2, agglomerative nesting analysis (AGNES) with the Ward linkage method [55,56] was used to hierarchically cluster turtles according to the relative FPI contribution of their eight anatomical regions; according to this analysis, each turtle was assigned to an “FP anatomical group”. ANOVA (with Tukey post-hoc tests) was used to compare FPI among individuals assigned to the three FP anatomical groups. Furthermore, AGNES was used to hierarchically cluster anatomical regions according to their relative contribution to the FPI of the studied turtles. Multinomial logistic regression (MLR) models were then used to determine whether the FP anatomical group of a turtle (factor variable: A, B, C) could be predicted by the following variables: period (factor: 2010–2014, 2018–2022), context (factor: live capture, live rescue, beach carcass), CCL (cm), BMSR (percentage), protected area (logical), bay (logical), distance to nearest river mouth (km), distance to nearest metallurgical plant (km), turtle stranding density within 10 km (integer), mean human population density within 10 km (inhabitants/km^2^), mean CHL within 10 km (mg/m^3^), mean SSS within 10 km (‰), mean annual range of SSS within 10 km (‰), mean SST within 10 km (°C), and mean annual range of SST within 10 km (°C).

The stepwise procedure informed by the Akaike Information Criterion [57] was used to select the best BLR and MLR models. The Cragg & Uhler pseudo-R^2^ index [57] was used to evaluate goodness-of-fit and the likelihood ratio test was used to determine the significance of each variable. Significance level was 0.05 for all tests. Because some individuals did not have data for some variables, the effective sample size for the BLR model produced with dataset 1 was 1785 turtles; this was not considered problematic because selectively excluding variables with incomplete sample size did not significantly change which variables were selected for the final model.

## Results

### Dataset 1: Fibropapillomatosis tumor prevalence

**Fig 2:**
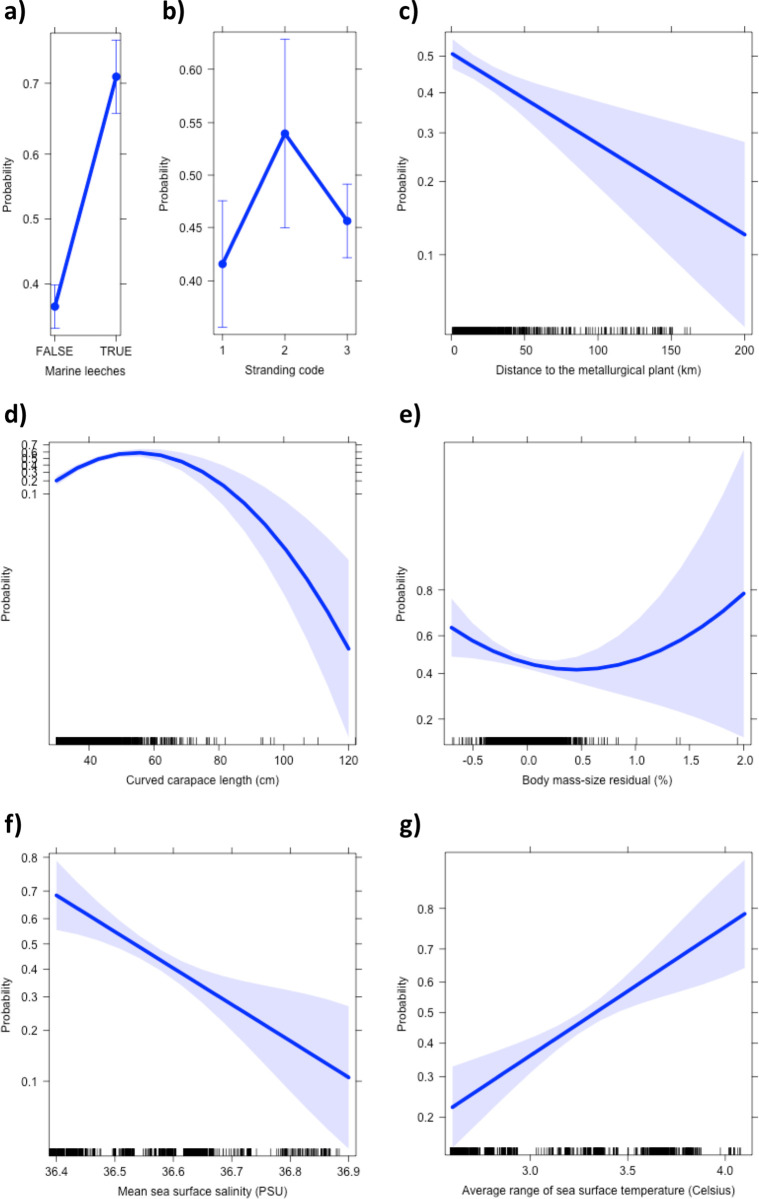
Effect of study variables in a binomial logistic regression model to predict the occurrence of FP in green turtles (*Chelonia mydas*, n = 1785) sampled at Espírito Santo, Brazil. Legend: a) marine leeches, b) stranding code, c) distance to nearest metallurgical plant, d) curved carapace length, e) body mass-size residual, f) mean sea surface salinity, and g) annual range of sea surface temperature.

External tumors consistent with FP were recorded in 40.9% (828/2024) of green turtles stranded in reasonable condition of preservation (live individuals and carcasses of codes 2 and 3) along the coast of Espírito Santo from 2018 to 2021. The best BLR model to predict the presence/absence of FP tumors comprised seven variables (Table 1 and Fig 2; Cragg & Uhler pseudo-R^2^ = 0.215): (a) marine leeches, (b) stranding code, (c) distance to nearest metallurgical plant, (d) CCL, (e) BMSR, (f) mean sea surface salinity, and (g) annual range of sea surface temperature. Average CCL was 41.51 ± 7.35 cm (mean ± SD) for turtles with FP and 39.86 ± 9.33 cm for turtles without FP. Average BMSR was –2.47% ± 20.79% for turtles with FP and 1.63% ± 20.95% for turtles without FP.

**Table 1:** Coefficient estimates, standard error (S.E.), *t*-value, odds ratio (O.R.) and *P-*value of variables in the binomial logistic regression model to predict the presence of FP tumors in green turtles (*Chelonia mydas*, n = 1785) sampled at Espírito Santo, Brazil.

Variable	Coef.	S.E.	*t*	O.R.	*P*
Intercept	199.279	61.187			
Marine leeches (ref. category: absence)	1.435	0.125	11.49	4.20	<0.001
Stranding code 2 (ref. category: code 1)	0.500	0.213	2.35	1.65	0.019
Stranding code 3 (ref. category: code 1)	0.165	0.135	1.23	1.18	0.220
Curved carapace length (cm)	0.312	0.005	6.16	1.37	<0.001
Curved carapace length (cm), squared	–0.003	0.001	–5.38	0.99	<0.001
Body mass-size residual (%)	–0.615	0.288	–2.14	0.54	0.003
Body mass-size residual (%), squared	0.682	0.421	1.62	1.98	0.105
Distance to nearest metallurgical plant (km)	–0.010	0.003	–3.48	0.99	<0.001
Mean SSS (‰)	–5.832	1.708	–3.41	0.03	<0.001
Annual range of SST (°C)	1.710	4.189	4.083	5.53	<0.001

### Dataset 2: Anatomical distribution of fibropapillomatosis tumors

**Fig 3:**
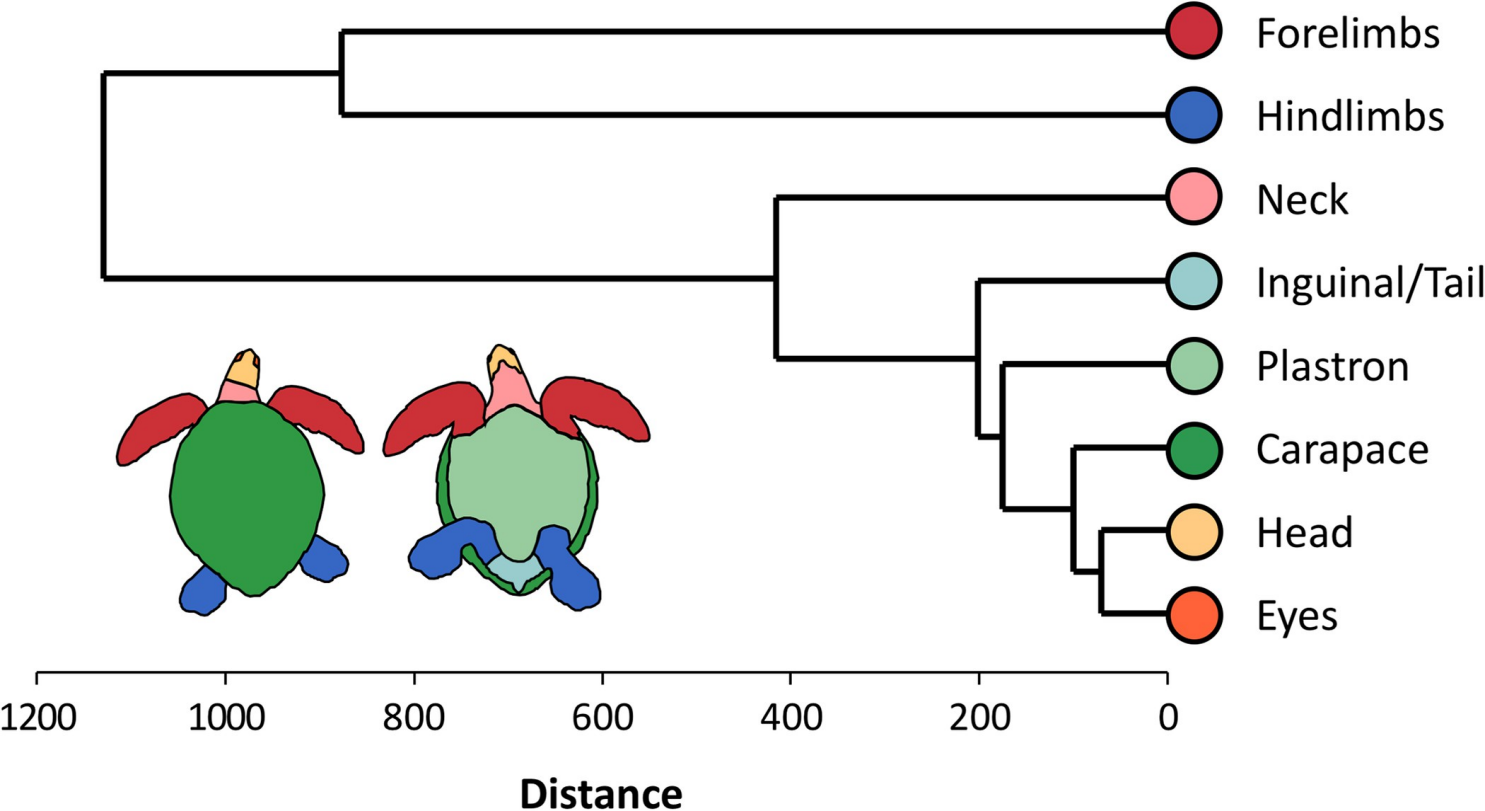
Dendrogram representing how anatomical regions were grouped by the agglomerative nesting analysis based on their relative FPI contribution in green turtles (*Chelonia mydas*, n = 271) with fibropapillomatosis sampled at Espírito Santo, Brazil.

**Fig 4:**
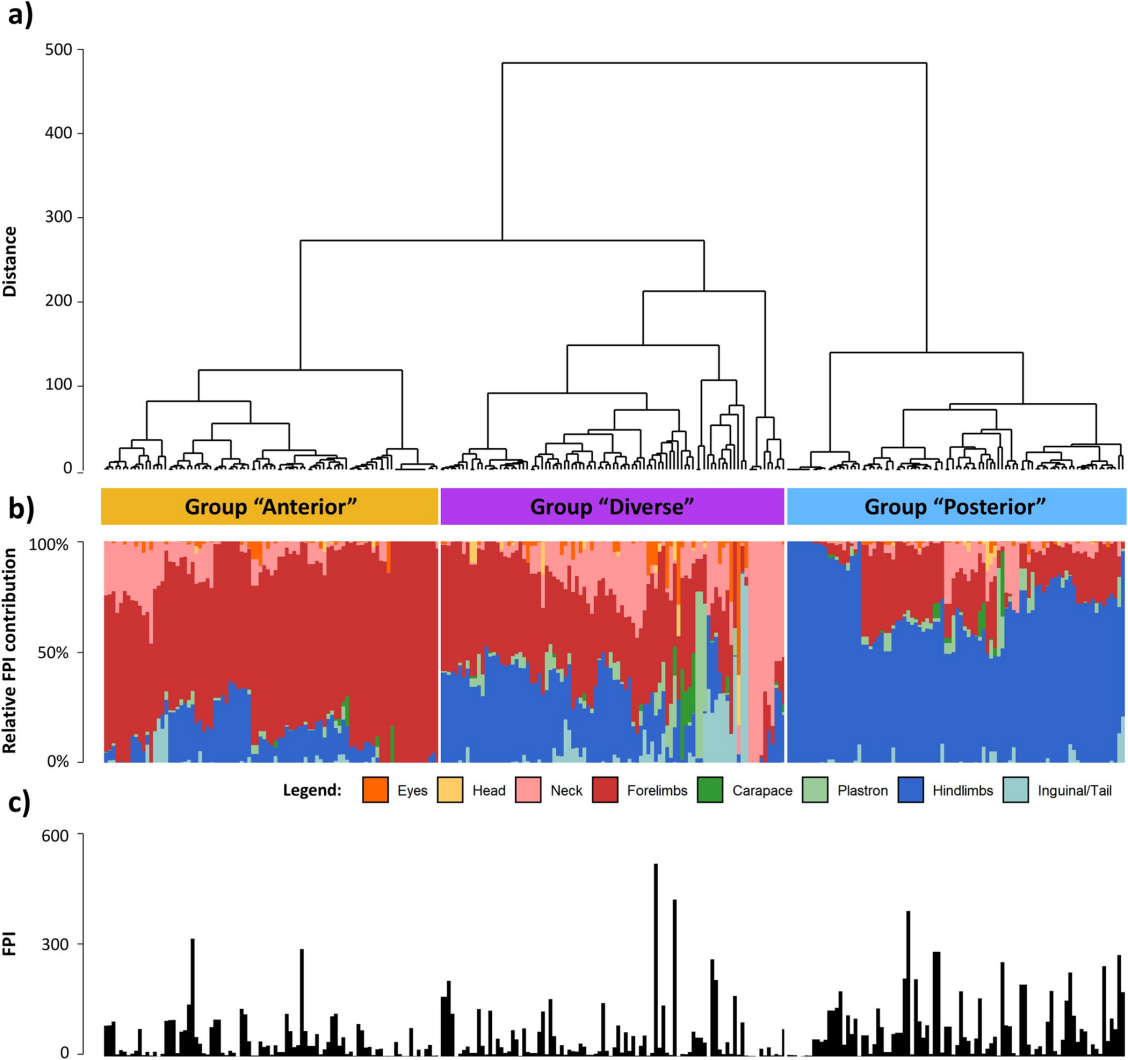
Classification of green turtles (*Chelonia mydas*, n = 271) with fibropapillomatosis (FP) sampled at Espírito Santo, Brazil, according to the anatomical distribution of their tumors. Legend: a) dendrogram representing how turtles may be classified into three FP anatomical groups based on the agglomerative nesting analysis of the relative FPI contribution of eight anatomical regions; b) stacked bar plots of the relative FPI contribution of these anatomical regions for each turtle; c) bar plots of the total FPI for each turtle.

**Fig 5:**
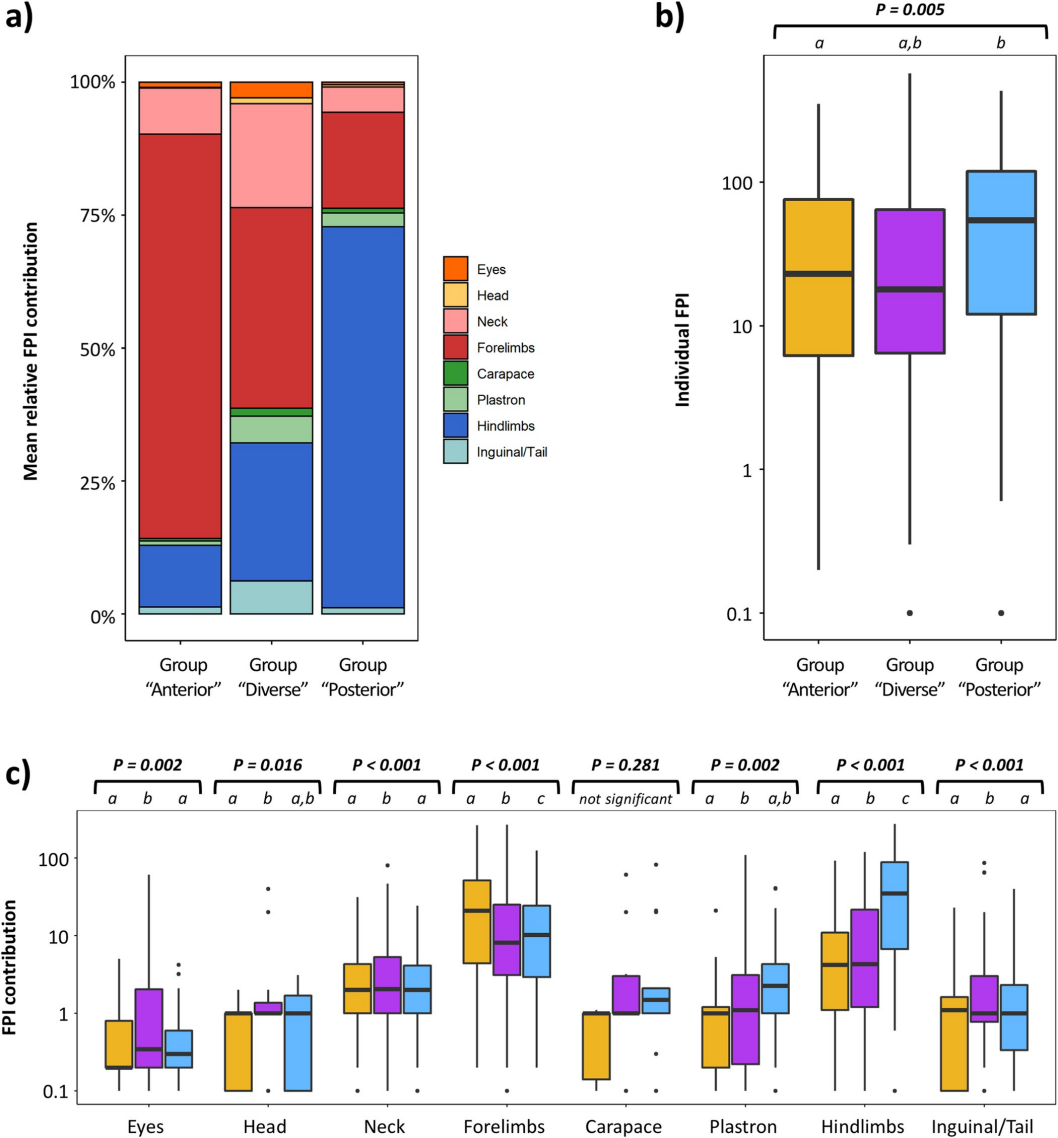
Summary of the clinical presentation of the fibropapillomatosis (FP) anatomical groups identified in green turtles (*Chelonia mydas*, n = 271) sampled at Espírito Santo, Brazil. Legend: a) bar plot of the average relative FPI contribution of each anatomical region across FP anatomical groups; b) boxplot of FPI across FP anatomical groups; c) boxplot of the FPI contribution of each anatomical region across FP anatomical groups. *P-values* above plots represent ANOVA test results, with italicized letters indicating groups with significant difference in post-hoc Tukey comparisons. Boxplot colors represent FP anatomical groups: Orange (group “anterior”), purple (group “diverse”), and blue (group “posterior”).

**Fig 6:**
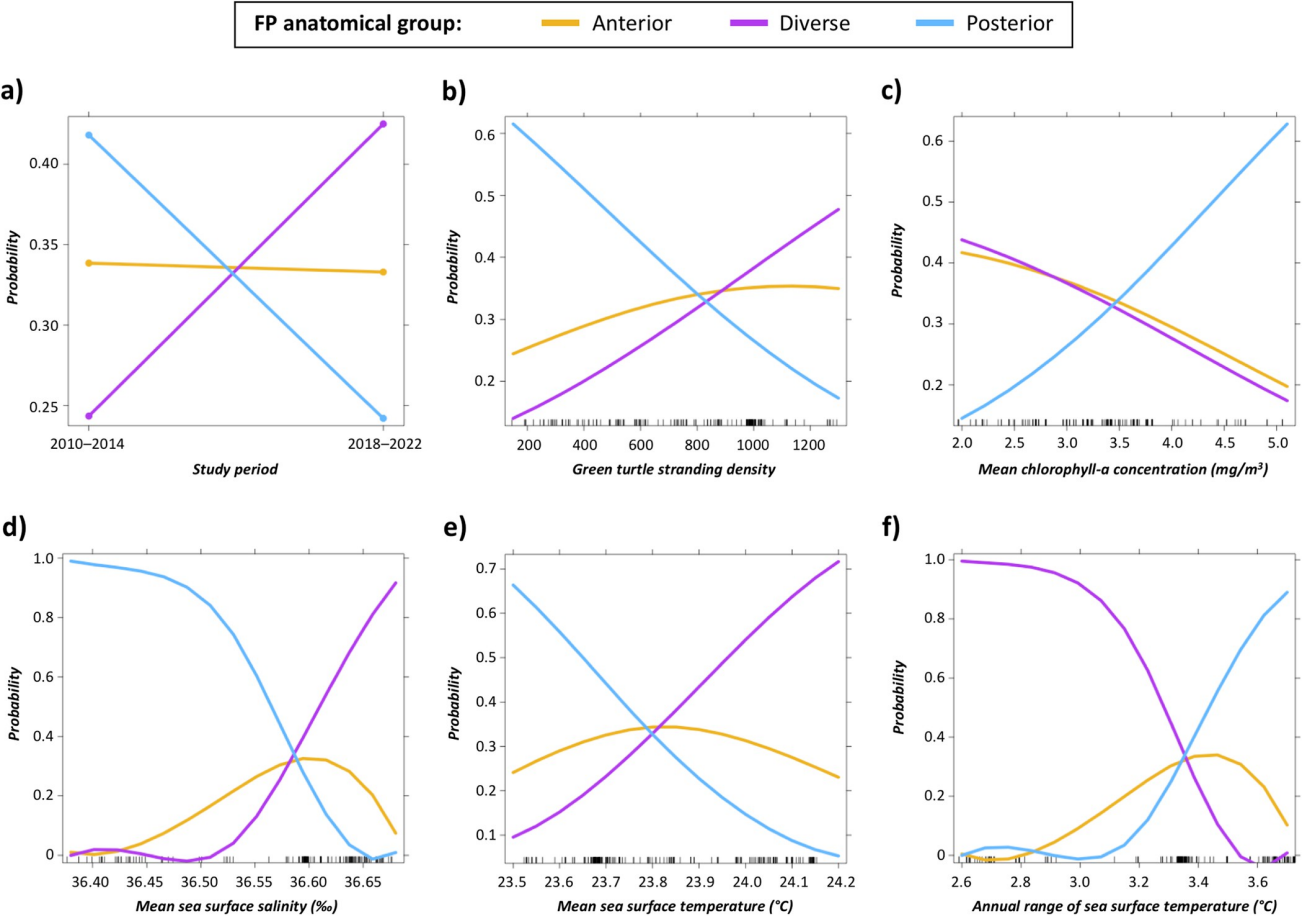
Effect of study variables in a multinomial logistic regression model to predict the FP anatomical groups in green turtles (*Chelonia mydas*, n = 271) sampled at Espírito Santo, Brazil. Legend: a) study period; b) green turtle stranding density; c) mean annual sea surface chlorophyll-a concentration; d) mean annual sea surface salinity; e) mean annual sea surface temperature; f) annual range of sea surface temperature.

Hierarchical clustering of anatomical regions revealed that the relative FPI contribution of anatomical areas in the axial body tended to covary (in decreasing order of similarity: eyes, head, carapace, plastron, inguinal region/tail, and neck) whereas the forelimbs and hindlimbs varied independently from these anatomical regions and from each other (Fig 3). Based on the relative FPI contribution of different anatomical regions, AGNES classified green turtles into three FP anatomical groups. Fig 4A presents the dendrogram representation of how turtles were hierarchically classified within these three groups, whereas Fig 4B and Fig 4C represent respectively the relative FPI contribution of different anatomical parts and the total FPI for each turtle. Fig 5A and 5B summarize the relative FPI contribution of different anatomical parts and the total FPI for each of the three groups; Fig 5C provides a breakdown of the FPI contribution from each anatomical part across the three groups. Turtles in group “anterior” had a greater relative FPI contribution from the forelimbs, whereas turtles in group “posterior” had a greater relative FPI contribution from the hindlimbs (Figs 4 and 5). On the other hand, turtles in group “diverse” presented a highly diverse anatomical distribution of tumors, with balanced relative FPI contributions from the forelimbs and hindlimbs, as well as some cases where FPI was primarily driven by tumors on the plastron or neck (Fig 5C). Fibropapillomatosis was generally more severe for turtles in group “posterior” compared to turtles in groups “anterior” and “diverse” (Table 2 and Fig 5B and 5C). The best MLR model to predict the FP anatomical group of turtles comprised six variables (Table 3 and Fig 6; Cragg & Uhler pseudo-R^2^ = 0.116): (a) study period, (b) green turtle stranding density, (c) mean sea surface chlorophyll-a concentration, (d) mean sea surface salinity, (e) mean sea surface temperature, and (f) annual range of sea surface temperature.

**Table 2:** Comparison of the fibropapillomatosis index (FPI) and Southwest Atlantic fibropapillomatosis score classification according to FP anatomical groups of green turtles (*Chelonia mydas*, n = 271) sampled at Espírito Santo, Brazil.

Group	FPI		FPS_SWA_			*N*
	Mean ± SD	Range	Mild	Moderate	Severe	
Anterior	46.3 ± 60.2	0.2–349.7	64%	28%	8%	89
Diverse	56.3 ± 92.3	0.1–571.9	65%	18%	16%	92
Posterior	84.9 ± 90.4	0.1–431.7	40%	34%	26%	90
Total	62.5 ± 83.8	0.1–571.9	56%	27%	17%	271

**Table 3:** Coefficient estimates, standard error (S.E.), *t*-value, odds ratio (O.R.) and *P*-value for the variables in the multinomial logistic regression model to predict the FP anatomical groups (relative to group “anterior”) in green turtles (*Chelonia mydas*, n = 271) sampled at Espírito Santo, Brazil.

Variable	Group “diverse”				Group “posterior”				*P*
	Coef.	S.E.	*t*	O.R.	Coef.	S.E.	*t*	O.R.	
Intercept	–1065.288	0			871.847	0			
Study period (ref. category: 2010–2014)	0.287	0.162	1.772	1.333	–0.265	0.161	–1.646	0.767	0.004
Green turtle stranding density (integer)	0.001	0.001	1.124	1.001	–0.001	0.001	–1.873	0.999	0.055
Mean CHL (mg/m^3^)	–0.057	0.017	–3.316	0.945	0.713	0.013	54.583	2.041	0.098
Mean SSS (‰)	0.287	0.007	40.206	1.321	–0.221	0.008	–28.462	0.801	0.004
Mean SST (°C)	0.029	0.011	2.726	1.030	–0.035	0.012	–2.907	0.965	0.004
Annual range of SST (°C)	–0.072	0.006	–12.511	0.930	0.064	0.007	9.480	1.066	0.002

## Discussion

In this study, we used a two-pronged approach to investigate the epidemiology of FP in green turtles in the Southwest Atlantic Ocean. Firstly, we analyzed a large dataset from a beach monitoring program covering ~400 km of coastline over a four-year period, wherein the presence or absence of FP tumors was systematically recorded (dataset 1). Because this dataset was produced through continuous daily beach surveys where all stranded live and dead sea turtles were recorded following standardized protocols, these results are not subject to the confusion associated with methodological constraints such as data gaps, varying temporal scales, and data capture methods, which has often been a limitation in FP epidemiological studies [58]. However, while this dataset provides an excellent opportunity to explore the individual and environmental variables associated with the prevalence of FP, it lacks details about the clinical presentation of the disease.

Secondly, we analyzed a smaller dataset from several research projects conducted over a subsection (~250 km) of the study area wherein detailed data was collected on the size and anatomical distribution of external FP tumors (dataset 2). This dataset comprises exclusively individuals that had FP, and therefore cannot be used to determine the factors driving the occurrence of this disease. However, it provides a valuable opportunity to investigate whether there are recurring patterns in FP tumor anatomical distribution and what individual and environmental variables may be associated with these patterns. The combination of these two approaches allows us a more comprehensive perspective on the epidemiology of FP in a high-prevalence tropical coastal environment, which may help us better understand why this emerging disease has become a significant threat to several green turtle populations worldwide.

### Factors determining the presence or absence of FP tumors (dataset 1)

Approximately 41% of individuals that stranded in the study area alive or as reasonably preserved carcasses showed external FP tumors, which is generally consistent with previous FP prevalence estimates for the study area [18,28,33–35]. However, because of the different methods used to survey FP prevalence over the years (beach surveys, intentional capture and dive surveys), it is not possible to ascertain the historical trends of this disease in the study area.

Marine leeches (*Ozobranchus* spp.) are considered the leading candidates for mechanical vectors of ChHV5 [7], given they can carry high viral DNA loads [59] and there is robust epidemiological association between FP and marine leeches [60,61]. Our results further corroborate that this association occurs at the study site, since stranded sea turtles with marine leeches were more than four times more likely (OR = 4.20) to present external FP tumors than those that stranded without marine leeches. It is worth noting that marine leeches are often found attached to the soft and highly vascularized skin within the folds and crevices of verrucous FP tumors, presumably benefiting from the reduced hydrodynamic drag and the soft skin surface [61]. In this context, there appears to be a synergism between marine leeches and ChHV5, with leeches acting as mechanical vectors for the ChHV5 and in turn ChHV5-induced tumors providing optimal microhabitat for leeches. Further studies are warranted to examine this hypothesis by determining the anatomical distribution of marine leeches in sea turtles with and without FP and evaluating whether the presence of FP tumors benefits the attachment, survival, and proliferation of marine leeches and whether this association between marine leeches and FP tumors occurs consistently across geographical regions. Additionally, it would be worth investigating whether the different clinical presentations of FP (smooth vs. verrucous tumors, size and anatomical distribution of tumors, surface ulceration, etc.) may affect the prevalence and distribution of marine leeches and, in turn, modulate their efficacy as vectors. It is also worth noting that different species of marine leeches may differ in their role in the epidemiology of ChHV5. *Ozobranchus branchiatus* is the most common in green turtles, whereas *Ozobranchus margoi* is more common in loggerhead turtles (*Caretta caretta*) and sporadically parasitizes green turtles where these hosts overlap in distribution [60,62]. Viral surveys suggest that *O*. *branchiatus* are more likely to encounter and remain on FP-positive hosts, and hence might play a more effective role as a vector of ChHV5 [60]. Understanding the epidemiological role of different marine leech species and how they interact with other environmental factors (e.g. climate-mediated changes in sea turtle and marine leech distribution, loss of cleaning stations due to collapse of reef systems, etc.) could therefore be valuable to improve our understanding of the impacts of FP and how they could be mitigated.

The stranding code was associated with the occurrence of FP, with tumors being more frequent in fresh carcasses (code 2) than in live animals (code 1); no such effect was detected for moderately decomposed carcasses (code 3). This could reflect a bias in how carcasses are obtained. Carcasses delivered to the beach monitoring program by the fishermen after having been retrieved from bycatch in fishing nets are more likely to be fresher than those found ashore. As a result, fresh carcasses have a higher proportion of anthropogenic interaction with fishery bycatch (23%) than do live animals (13%) and moderately decomposed carcasses (15%) (S2 Table). It is therefore possible that the higher prevalence of FP in fresh carcasses relates to the fact that this cohort comprised a greater proportion of bycaught individuals, thereby indirectly reflecting biases in capture effort at specific areas or depths. Alternatively, bycaught individuals could be less likely to present with other conditions that are negatively associated with FP.

Fibropapillomatosis tends to be most common in juvenile green turtles with CCL between 40 and 70 cm [26,28,31], which is thought to be related to the development of the immune system and may also reflect changes in diet and habitat use [7]. Most turtles in this study were relatively young (CCL predominantly between 30 and 50 cm), presumably having recently transitioned to the neritic habitat [63–65]. FP-affected turtles in this study tended to present with a slightly greater carapace length than FP-free turtles (on average, 1.6 cm greater), a pattern that has been consistently reported across the world [24,31,35,66]. Assuming a similar growth rate in FP-affected and FP-free turtles, as documented in previous studies [67], this suggests that the FP-affected turtles were on average slightly older than FP-free turtles in this cohort, possibly reflecting the time lapse associated with the onset and growth of FP tumors upon arrival to the neritic habitat. On the other hand, FP occurrence gradually decreased in turtles with CCL greater than 70 cm, which is consistent with tumor remission (as suggested by disease state modelling studies [68]), development of disease resistance, or death/removal of affected individuals.

The results of this study suggest that FP tends to be most common in turtles with abnormally low or abnormally high BMSR. In individuals with poorer body condition (as presumed from low BMSR), this could be interpreted that malnutrition could be either a cause (tumor development being enhanced by an impaired immune system in malnourished individuals) or a consequence of FP (tumors diverting nutritional resources or impairing the ability to forage). Interestingly, previous studies in the study area found that FP-affected turtles had similar or better body condition than FP-free turtles [32,33], and our results also point to this as a possibility. In these cases, it is worth considering that the tumors themselves can have a substantial mass, and thus it is possible that FP-affected turtles may have a lower proportion of muscular and visceral mass than FP-free turtles and yet their BMSR is falsely elevated by tumor mass. In this context, FP-affected turtles may have experienced an even lower availability of nutritional resources to meet their physiological needs than would be appreciated from BMSR. Future studies would be valuable to clarify if and how FP modulates the allocation of mass among different tissues, and how this reflects on individual nutritional status and physiology.

A recent study found that human population density was a significant predictor of FP prevalence in green turtles stranded along the coast of Florida, USA [69] and yet no such association was found in this study. Here, human population density did not present a statistically significant effect but proximity to metallurgical plants was positively associated with FP occurrence. Metallurgical plants in Espírito Santo are known to favor the aggregation of juvenile green turtles, with above-average FP prevalence at the warm water effluent discharge of these plants [18,33–35]. Warmer water is known to promote tumor growth and has been implicated in the seasonality of FP in the northern hemisphere [6,10], hence it is plausible that industrial warm water discharges enhance tumor growth. It is also plausible that metallurgical plants could indirectly enhance FP prevalence by impairing the immune system through increased exposure to trace elements or other pollutants [29], changes in marine plankton resulting in the release of biotoxins (see [70,71]) or in shifts in dietary amino acid profile [19,72] (but see criticism by Work and colleagues [21]). Surveys of water, suspended matter, sediment, and mussels at Vitória and Espírito Santo Bays (where there is substantial effluent discharge from metallurgical plants, in addition to high human population density and maritime port activity) have shown elevated levels for some trace elements (Ag, As, Co, Cr, Cu, Mg, Ni, Pb, Zn). Although elevated, these levels were below acute toxicity and legal thresholds [73–75], with the exception of arsenic, for which the high levels have been attributed to natural sources [75]; it remains unclear whether some of these trace elements could play a role in FP tumor development, as has been debated in the past [76–78]. Alternatively, a simpler explanation would be that the aggregation of juvenile green turtles at these warm water discharges favors ChHV5 transmission simply due to the high local density of turtles, perhaps potentiated by a temperature-mediated enhancement in the growth and proliferation of marine leeches. Further studies on the diet, behavior, parasites and trace element concentrations in the tissues of green turtles at these aggregation sites may help clarify how industrial warm water discharges influence the epidemiology of FP.

The occurrence of FP was negatively associated with the mean SSS and positively associated with the annual range of SST. Marine leeches from sea turtles are known to prefer high salinity environments (>30‰) [79], with freshwater baths being used to kill marine leeches at sea turtle rehabilitation facilities [61]. We would thus have expected a positive association between mean SSS and FP, which was not the case. Alternatively, because it has been speculated that green turtles may visit brackish waters to control marine leeches through hypotonic shock [61], we would have expected a negative association between FP and the annual range of SSS or the proximity to river mouths, and yet our results did not corroborate this either. It is plausible that we did not detect these indirect associations we expected between FP and salinity because they were overshadowed by the stronger and more direct effect from the presence/absence of marine leeches in the statistical model. Interestingly, a recent study found that salinity (0–5 m water depth, as estimated by ocean models) was positively associated with FP prevalence in green turtles stranded along the coast of Florida but was negatively associated with FP prevalence in green turtles stranded along the coast of Texas, USA [69]. These conflicting results highlight our poor understanding of how seawater salinity modulates the epidemiology of FP, suggesting that complex interactions might be involved. With regards to sea surface temperature, seasonal variations in temperature have been linked to the epidemiology of FP [6,10]. and a positive association was found between SST and FP prevalence in green turtles collected in-water in Florida [69]. However, it should be noted that the SST in the study area can be considered amenable (annual mean between 22 and 26°C) and with a narrow seasonality (annual range lower than 4°C) compared to that of temperate regions or upwelling systems also inhabited by sea turtles. Hence, we did not expect natural variation in SST to play as determinant a role in driving FP epidemiology when compared to the SST hotspots produced by metallurgical plants (up to 35°C [35]). Considering the lack of a clear biological explanation and that both mean SSS and annual range of SST follow marked latitudinal gradients along the study area (see S1 File) and may be correlated to environmental factors other than those considered in this study (e.g. nutrient and chemical pollution or discharge by rivers, water column stratification dynamics, phytoplankton communities, etc.), it seems plausible that the statistical effects detected in this study reflect hidden variables (variables that are geographically correlated but which were not evaluated in this study). In order to better discern whether seawater salinity and temperature are actually driving FP epidemiology or are simply serving as proxies for hidden variables, it would be useful to replicate this analysis over broader geographic scales, to verify if the same statistical associations are consistently observed across coastal systems and oceans.

### Factors determining the anatomical distribution of FP tumors (dataset 2)

Work and Balasz [46] were the first to propose a system to categorize FP tumors by size, developing a semi-quantitative score that has been widely used to classify the severity of FP in green turtles [22,28,34,44,80–83]. More recently, Rossi and colleagues [8] proposed an objective index to quantify the severity of FP based on the number and size of tumors, the fibropapillomatosis index (FPI), as well as a standardized approach to categorize the anatomical distribution of tumors. In this study, we expand upon this foundation by employing hierarchical clustering analysis to explore if there are recurring patterns in the anatomical distribution of FP tumors, and then determining which individual and environmental variables might be associated with these patterns.

We found that there was considerable variability in the contribution of anatomical areas in relation to the number and size of FP tumors. The relative FPI contribution of the axial body tended to covary, whereas the relative FPI contribution of the forelimbs and hindlimbs appeared to vary independently from each other and from the remainder of the body, possibly reflecting histological or physiological particularities of these anatomical regions. This, in turn, translated into the green turtles with FP in this study showing three relatively distinct patterns of anatomical distribution of tumors. Turtles in group “anterior” had more numerous/larger tumors on the forelimbs, turtles in group “posterior” had more numerous/larger tumors on the hindlimbs, and turtles in group “diverse” had a diverse anatomical distribution of tumors. The well-defined structure of the dendrogram produced by the agglomerative nesting analysis (Fig 4A) confirms that these are distinct patterns of anatomical distribution, and not just a post-hoc categorization of an otherwise stochastic distribution. The possibility that the FP anatomical groups represent sequential stages of disease development (e.g. group “posterior” representing a later stage of the disease) is also contradicted by the fact that each FP anatomical group has a greater FPI both in relative and absolute terms (see Fig 5C: group “anterior” has the greatest FPI for forelimbs, group “posterior” has the highest FPI for hindlimbs, and group “diverse” has the greatest FPI for eyes, neck and inguinal/tail region). Hence, these results verify that the three FP anatomical groups do indeed represent distinct anatomical patterns of tumor growth.

The biological mechanisms driving these anatomical patterns, however, are unknown. Experimental infection studies showed that the tumors initially tended to develop primarily at the inoculation/scarification site [10], hence the finding of distinct FP anatomical groups could be interpreted as indicative of different sites/routes of ChHV5 exposure. It has also been noted that marking green turtles with flipper metal tags will sometimes trigger the development of FP tumors at the tag perforation site [25,67,84]. This suggests that the tag injury serves as a point of entry for ChHV5 or that the local inflammatory process elicited by the tag injury can activate latent ChHV5 infections, and has led to the recommendation that flipper tagging should be avoided when possible at FP endemic areas [25]. In this context, it is plausible that different routes of exposure to ChHV5 (e.g. marine leeches, trauma or epibiont-related injury, ingestion) could be associated with different points of entry of the virus, resulting in different anatomical patterns of tumor development. Contradicting this interpretation, however, it should be borne in mind that studies have shown that viral DNA can be detected in non-tumored skin of affected and unaffected turtles [14–17], which suggests instead that point of entry of the virus might not be a significant determinant of tumor development and growth. For this reason, we favor the interpretation that the differences in anatomical distribution of tumors observed in this study reflects other processes related to the triggering of tumor development or physiological or immune-mediated processes modulating tumor growth. In this context, further studies on how physiological and immunological factors modulate FP presentation, severity and presentation would be useful to clarify the epidemiology of this disease.

The probability of an FP-affected turtle being classified into a given FP anatomical group was predicted by the following variables: study period, green turtle stranding density, mean CHL, mean SSS, mean SST, and annual range of SST. Compared to the turtles sampled in the earlier study period (years 2010–2014), turtles sampled more recently (years 2018–2022) had a smaller likelihood of being classified in the “posterior” group and a greater likelihood of being classified in the “diverse” group, whereas the likelihood of being classified in the “anterior” group remained essentially unchanged. The results therefore suggest a historical change in the anatomical patterns of FP, which is something that was not evaluated in previous longitudinal studies on the epidemiology of this disease.

The probability of a turtle being classified in the “posterior” group (relative to the “anterior” group) was positively associated with annual range of SST and mean CHL, and negatively associated with mean SSS, mean SST and green turtle stranding density. The opposite associations were found for the “diverse” group. These findings suggest that these FP anatomical groups may have been associated with different habitats. Furthermore, it is interesting to note that the two environmental variables (mean SSS and annual range of SST) which had been identified as predictors of FP presence or absence (dataset 1) were also included in the model for FP anatomical distribution, which suggests that these two variables are indeed meaningful predictors of FP epidemiology. While it is possible that such environmental variables could have a direct effect on tumor development (e.g. temperature regulating tissue metabolism and growth rate), it seems more likely that these variables are proxies that represent different habitat types with features such as seasonal freshwater discharge by rivers, nutrient and chemical pollution by agriculture, industries and urban settlements, water column stratification in bays and other sheltered environments, etc. These features may translate into changes in the plankton and algal communities, biological productivity, marine leech prevalence, etc. which will, in turn, modulate the epidemiology and anatomical distribution of FP tumors. In this context, it is possible that significant epidemiological trends may be missed by studying FP as one cohesive disease presentation. Instead, interpreting FP as a mosaic of multiple anatomical presentations that are modulated by different–perhaps even contradictory–biological mechanisms may provide additional insight to understanding this disease and explaining why it varies considerably in epidemiological behavior and anatomical presentation among regions of the world.

It is noteworthy that turtles in group “posterior” presented generally higher FPI values (Table 2 and Fig 5B), indicating that their FP tumors were larger and/or more numerous, and this was mainly due to the FPI contribution from the hindlimbs (Fig 5C). Green turtles rely primarily on their forelimbs to swim, keeping their hindlimbs tucked in and pointing back to reduce drag [85,86]. It is plausible that the mechanical stress associated with drag poses a constraint to tumor growth rate, with tumors at anatomically sheltered sites such as the hindlimbs showing increased growth. Alternatively, it may be that FP tumors on hindlimbs tend to have a lesser impact on turtle mobility, which would explain why turtles in the “posterior” group were able to cope with more severe FP before succumbing or stranding. If certain FP anatomical groups are more impactful or associated with poorer prognosis, these presentations could be targeted by conservation initiatives by addressing their inciting causes. This could also allow rehabilitation centers to prioritize caring for animals with anatomical presentations with a more favorable prognosis. However, it is worth bearing in mind that this study utilized FPI which measures severity in terms of tumor mass, which may not necessarily be correlated with health impacts. Studies on the hydrodynamic drag and other constraints (e.g. limb mobility and propulsion, vision, foraging, etc.) posed by FP tumors at different anatomical areas would therefore be valuable to improve our understanding of how this disease affects fitness and health of sea turtles.

### Study limitations

Because this study was primarily based on data from beach surveys, as opposed to dive surveys, our findings could have been distorted by hidden variables affecting the probability of death or stranding of turtles in the study area. FP has direct and indirect negative health effects and may ultimately contribute to the stranding or death of affected turtles [11,24], hence beach surveys may overestimate the prevalence and severity of this disease. Another limitation to consider is that this study relies on the assumption that the environmental characteristics of the stranding site reflect the conditions under which FP development was triggered or modulated. But if FP-affected turtles were to show different habitat use compared to FP-free turtles, this would imply that the environmental factors identified in this study are not determinant of FP development, but rather are descriptive of the environments sought by turtles coping with FP. Studies on the epidemiology of FP using data collected through dive surveys and mark-recapture studies could help clarify these aspects and evaluate whether FP affects habitat use by sea turtles.

Another limitation is that we did not determine the origin of the green turtles in this study. Although Ascension Island is the primary source of juvenile green turtles in the study area, minor contributions from Trindade Island and western Africa are also thought to occur [4]. If genetic differences among green turtles from these nesting sites modulate FP susceptibility or anatomical presentation, as has been suggested to occur for herpesvirus infections in other hosts [87], this could have affected our results. Furthermore, this study relied on the assumption of viral homogeneity. Although it would seem that the genetics of ChHV5 do not influence the probability of tumor manifestation [88], it has been proposed that viral variants may differ in disease anatomical presentation [59]. At least two ChHV5 variants are known to occur in the study area [44], hence if these variants show differences in pathogenicity or virulence this could have influenced the anatomical patterns found in this study. Future studies examining how host and viral genetics influence the development and clinical presentation of FP tumors would therefore be valuable to improve our understanding of this disease. Additionally, it should be noted that a recent study proposed that FP might result from dual infection by ChHV5 and a papillomavirus [23]. This hypothesis needs to be further investigated and, if confirmed, epidemiological studies of FP would require more complex models since these two viruses might respond differently to individual and environmental variables.

## Conclusions

This is the first study to demonstrate that there are distinct patterns of anatomical distribution of FP tumors in green turtles that may be associated with different environmental variables. Because this study was based on a limited geographic area (relative to the pantropical distribution of green turtles), where the environmental variables have limited range of variation and are correlated across overlapping spatial gradients, it would be premature to draw broad conclusions about the epidemiology of FP in green turtles. Our results do, however, point at the possibility that FP may have different anatomical presentations that are not necessarily driven by the same epidemiological factors. If confirmed, this would imply a paradigm shift where rather than being perceived as a single entity, FP should be seen as a mosaic of distinct anatomical patterns (at least three, perhaps more to be described) that must be considered separately in order to untangle the disease’s epidemiology. In this conception, ChHV5 infection would remain the cornerstone for the neoplastic process, but the observed patterns of tumor growth and anatomical distribution would be determined by other individual or environmental cofactors, which would ultimately determine the epidemiology and health outcomes of this disease.

## Supporting information

S1 File Maps of environmental variables in the study area: (a) landmarks mentioned in text and green turtle stranding density (shaded areas), (b) rivers (lines) and river mouths (circles), (c) bays, (d) protected areas, (e) metallurgical plants, (f) human population density, (g) mean annual sea surface chlorophyll-a concentration, (h) mean annual sea surface salinity, (i) annual range of sea surface salinity, (j) mean annual sea surface temperature, and (k) range of sea surface temperature.
(PDF)

S1 Table Stranding code classification for sea turtles (translated from Petrobras 2019 [45]). (PDF)

S2 Table Frequency of external FP tumors and evidence of bycatch according to the stranding code. (PDF)
