# Supplementary material for: Exploring the relationship between environmental drivers and the manifestation of fibropapillomatosis in green turtles (Chelonia mydas) in eastern Brazil
Source: PLoS One. 2023 Aug 24;18(8):e0290312. doi: 10.1371/journal.pone.0290312 (PMC10449228; doi:10.1371/journal.pone.0290312)
Supplement: S1 File — Maps of environmental variables in the study area: (a) landmarks mentioned in text and green turtle stranding density (shaded areas), (b) rivers (lines) and river mouths (circles), (c) bays, (d) protected areas, (e) metallurgical plants, (f) human population density, (g) mean annual sea surface chlorophyll-a concentration, (h) mean annual sea surface salinity, (i) annual range of sea surface salinity, (j) mean annual sea surface temperature, and (k) range of sea surface temperature. (PDF) [file pone.0290312.s001.pdf]

S1 File. Maps of environmental variables in the study area: (a) landmarks mentioned in text and green turtle stranding density (shaded areas), (b) rivers (lines) and river mouths (circles), (c) bays, (d) protected areas, (e) metallurgical plants, (f) human population density, (g) mean annual sea surface chlorophyll-a concentration, (h) mean annual sea surface salinity, (i) annual range of sea surface salinity, (j) mean annual sea surface temperature, and (k) range of sea surface temperature.

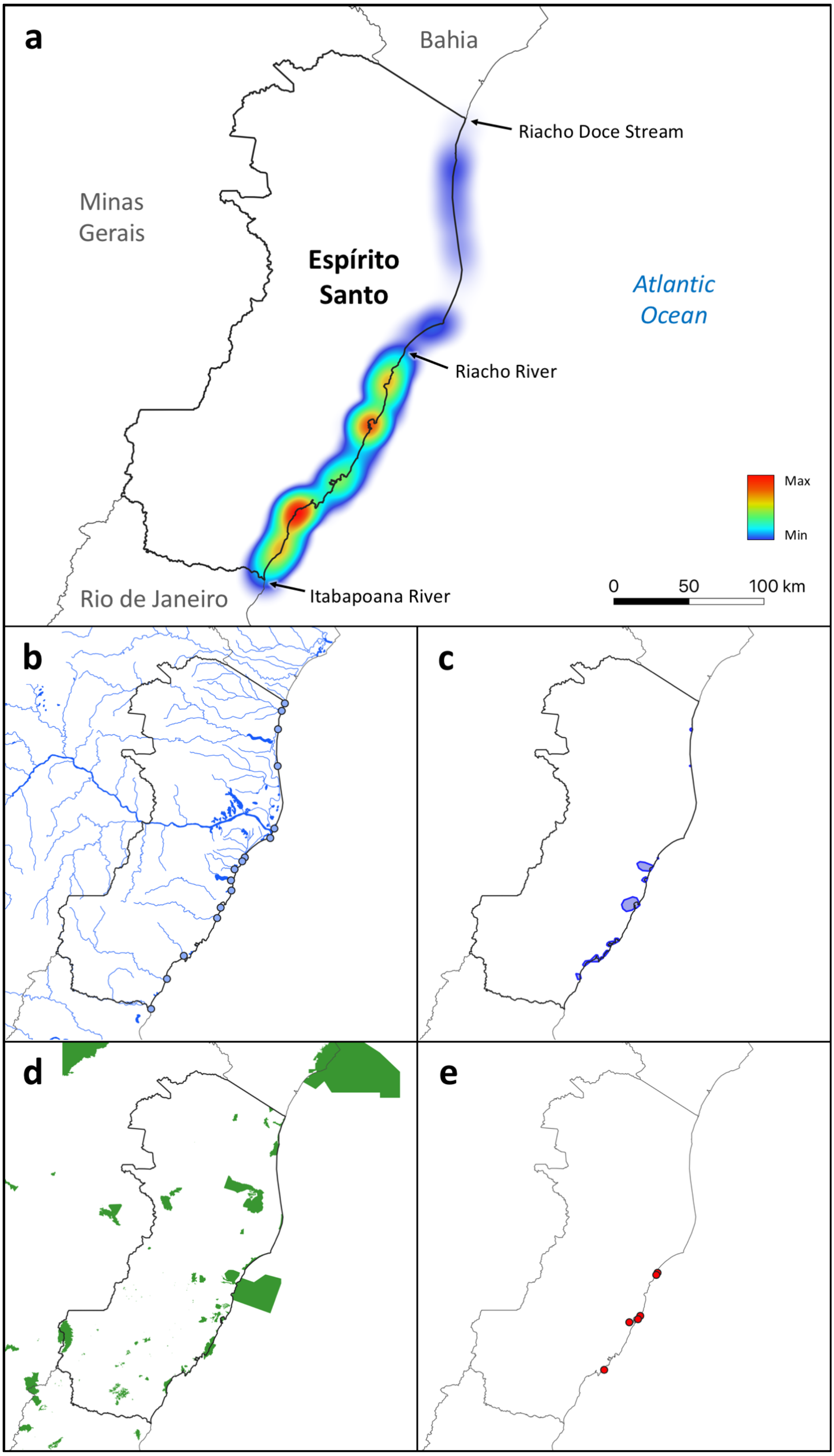

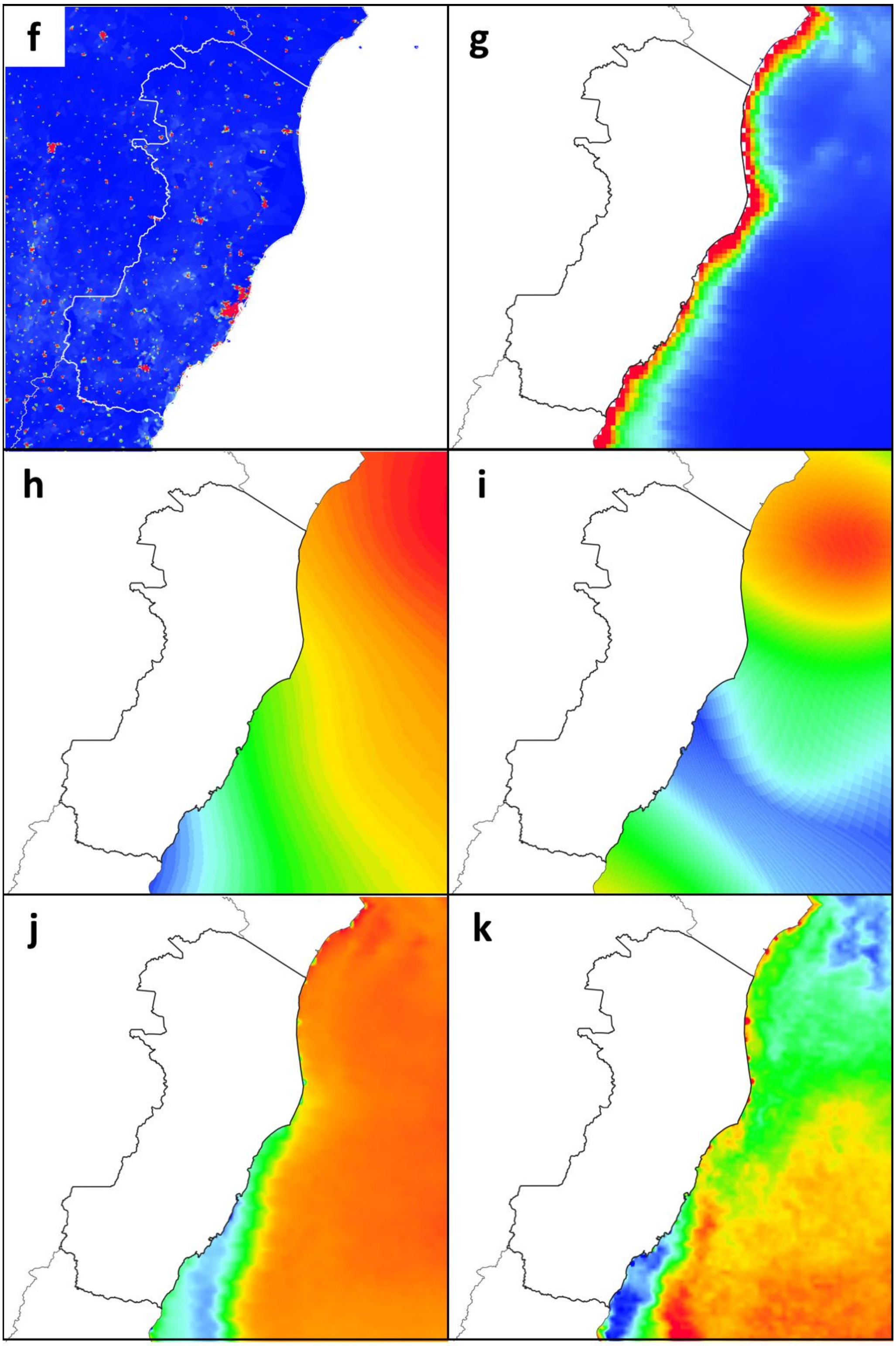

	(f) Human population density	(g) Mean sea surface chlorophyll-a concentration	(h) Mean sea surface salinity	(i) Annual range of sea surface salinity	(j) Mean sea surface temperature	(k) Annual range of sea surface temperature
Max	>1000 /km^2	3.40 mg/m^3	37.1 ‰	1.3 ‰	26.3 °C	4.5 °C
Min	1 /km^2	0.05 mg/m^3	36.3 ‰	0.6 ‰	23.0 °C	2.5 °C
