# Supplementary material for: Exploring the relationship between environmental drivers and the manifestation of fibropapillomatosis in green turtles (Chelonia mydas) in eastern Brazil
Source: PLoS One. 2023 Aug 24;18(8):e0290312. doi: 10.1371/journal.pone.0290312 (PMC10449228; doi:10.1371/journal.pone.0290312)
Supplement: S1 Table — (PDF) [file pone.0290312.s002.pdf]

**S2 Table.** Stranding code classification for sea turtles (translated from Petrobras 2019 [45]).

| Code | Status | Characteristics |
|---|---|---|
| 1 | Live animal | |
| 2 | Fresh carcass | External: lifelike appearance, little action of scavengers, firm skin, without changes in skin color.<br>Internal: firm musculature and fat, intact organs, intestine with small amount of gas. |
| 3 | Moderate decomposition | External: skin with mild hemolytic tint, protrusion of cloaca, slightly swollen neck, dehydrated eyes.<br>Internal: organs dyed by hemolytic and biliary imbibition, preserved architecture and consistency of organs and viscera, intestines dilated by gas. |
| 4 | Advanced decomposition | External: skin with marked hemolytic tint, presence of liquid pockets/blisters, obvious swelling of the neck, detachment of plates, evidence of carcass predation by scavengers, strong odor.<br>Internal: soft or liquefied fat, loss of architecture and consistency normal functioning of organs, organs are generally liquefied or with gas bubbles. |
| 5 | Mummified carcass or skeletal remains | |
