# Supplementary material for: Exploring the relationship between environmental drivers and the manifestation of fibropapillomatosis in green turtles (Chelonia mydas) in eastern Brazil
Source: PLoS One. 2023 Aug 24;18(8):e0290312. doi: 10.1371/journal.pone.0290312 (PMC10449228; doi:10.1371/journal.pone.0290312)
Supplement: S2 Table — (PDF) [file pone.0290312.s003.pdf]

S3 Table. Frequency of external FP tumors and evidence of bycatch according to the stranding code.

Stranding code	Turtles examined	Turtles with FP tumors	% with FP tumors	Turtles with evidence of bycatch	% with evidence of bycatch
1	453	187	41.3%	59	13.0%
2	158	78	49.4%	36	22.8%
3	1413	563	39.8%	205	14.5%
4	3796	548	14.4%	147	3.9%
5	935	37	4.0%	17	1.8%
